# Acquired methemoglobinemia induced by indoxacarb poisoning: a case report

**DOI:** 10.11604/pamj.2024.47.92.34766

**Published:** 2024-02-27

**Authors:** Dhouha Ben Braiek, Rania Hidri, Chaima Kaabi, Hend Zorgati, Imen Mighri, Rahma Ben Jazia, Ameni Kacem, Jihene Ayachi

**Affiliations:** 1 Medical Intensive Care Unit, Ibn El Jazzar University Hospital, Kairouan, Tunisia,; 2 Pulmonology Department, Ibn El Jazzar University Hospital, Kairouan, Tunisia

**Keywords:** Acquired methemoglobinemia, indoxacarb poisoning, case report

## Abstract

Indoxacarb, a large-spectrum non-organophosphorus oxadiazine insecticide, is broadly used in farming whose mechanism of action is the blockage of voltage-gated sodium channels of insects. There is restricted data on human poisoning. We report a case of an 18-year-old male patient without comorbidities presented with unconsciousness and cyanosis after the intentional ingestion of indoxacarb in a suicide attempt. Methemoglobinemia was clinically suspected and was successfully treated after methylene blue injection, associated with supportive and symptomatic management. This case emphasizes the importance of considering methemoglobinemia after indoxacarb ingestion in addition to its early recognition and timely injection of methylene blue which led to complete recovery without sequelae.

## Introduction

Agricultural insecticides have become a common household item in farming areas. They have been frequently used for self-poisoning because of their easy accessibility, and they are a significant cause of morbidity and mortality all over the world [1]. Many solvents used in insecticide formulations can cause human toxicity and may result in methemoglobinemia which is a life-threatening condition causing a decreased ability of hemoglobin to deliver oxygen [1]. Indoxacarb is a non-organophosphorus oxadiazine insecticide, that results in nerve function impairment and death of insects in a couple of hours. However, there is restricted information about its effects after human ingestion [2]. A review of the literature revealed few cases recorded regarding indoxacarb poisoning in humans and its complications such as methemoglobinemia around the globe, but there were none from Tunisia. Here, we present a case of acquired methemoglobinemia occurring after intentional indoxacarb ingestion during a suicidal attempt.

## Patient and observation

**Patient information:** an 18-year-old male patient, with no medical history, was brought to the emergency department (ED) by his family one hour after deliberate ingestion of 200 mL of pesticide containing indoxacarb in a suicide attempt.

**Clinical findings:** on arrival in the ED, he was drowsy, and he had a Glasgow Coma Scale (GCS) of 13/15, with bilateral mild-dilated reactive pupils. His vital signs were stable; he had no dyspnea with an oxygen saturation (SpO_2_) of 95% in ambient air, a blood pressure (BP) of 110/70 mmHg, a heart rate (HR) of 80 beats/min, and a body temperature of 36°C. The electrocardiogram (ECG) revealed normal sinus rhythm, and the chest X-ray showed no particular abnormality. After monitoring, he received gastric lavage. One hour later, the patient vomited two times, and he became deeply cyanotic, with a respiratory rate of 30c/min and SpO_2_ dropped to 80% despite receiving 15L/min of oxygen via a high-concentration oxygen mask indicating severe hypoxemia. The conscious state was altered, and he became unresponsive with GCS of 8/15 then the appearance of generalized fasciculations and muscle tremors. He had a BP of 100/40 mmHg and HR of 110 bpm.

**Figure 1:**
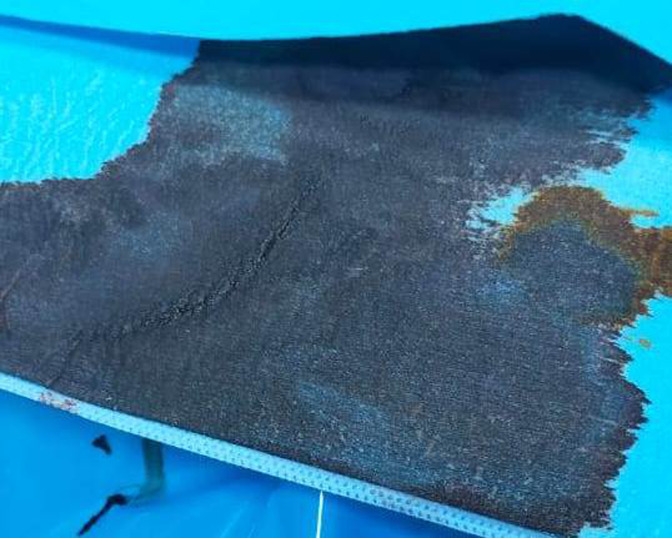
chocolate-brown colour of the patient's blood in the sterile field after placing a femoral central venous catheter due to methemoglobinemia

**Timeline of current episode:** in front of the worsening of his neurological and respiratory states, he was promptly intubated and put on assisted invasive mechanical ventilation with a fraction of inspired oxygen (FiO_2_) of 100%. In post-intubation, the patient presented a cardiovascular collapse (BP 80/40 mmHg and HR 120 b/m) requiring vasoactive drugs (0.5mg/h of norepinephrine), then transferred to our medical intensive care unit (ICU). Upon ICU arrival, the patient was under sedation, invasive mechanical ventilation, and norepinephrine, he had remarkable central cyanosis and the SpO_2_ did not exceed 80% despite FiO_2_ of 100% contrasting with normal respiratory exam. The patient´s blood colour was abnormally muddy brown (Figure 1) associated with dark orange urine.

**Diagnostic assessment:** laboratory analysis revealed: a white blood cell count of 23000/μL, hemoglobin: 13.6 g/dL, platelets: 273000/μL, alanine aminotransferase 45 IU/L, creatinine 48 μmol/L, sodium 137 mmol/L and potassium 4.26 mmol/L. Arterial blood gas analysis (ABG) on FiO_2_ 100% revealed pH 7.47, PaO_2_ 450 mmHg, PaCO_2_24 mmHg, HCO_3_ 17.3 mmol/L, and arterial oxygen saturation (SaO_2_) 100%. The SpO_2_ was only 85%. A chest CT scan was performed and showed no abnormality.

**Diagnosis:** methemoglobinemia was suspected considering the cyanosis, low SpO_2_ concomitant with disproportionately high PaO_2_ on ABG analysis, and muddy brown colour of blood. This could be secondary to indoxacarb poisoning. A blood sample was analyzed to determine the methemoglobin (MetHb) level, confirmed a level of 12.5% (reference range: <3%).

**Therapeutic interventions:** intravenous methylene blue was administered at a dosage of 1.5 mg/kg in 100 ml of normal saline for 30 minutes. Within two hours, the patient showed gradual signs of spectacular clinical recovery. The cyanosis has disappeared, and his SpO_2_ gradually improved to 92 - 94% then 100%. The ABG analysis revealed pH 7.36, PaCO_2_ 32, PaO_2_ 340, HCO_3_ 18.1, and SaO_2_ 100% on FiO_2_ of 25% three hours later.

**Follow-up and outcome of interventions:** the patient's respiratory and hemodynamic states were progressively improved, and he was weaned off vasopressor and mechanical ventilation. He was extubated on day three and put on a nasal cannula to maintain an efficient gas exchange. Supplemental oxygen was then discontinued on day four, with SpO_2_ of 97% in ambient air. The methemoglobin level measured after 12 hours of administration of methylene blue showed an important reduction to 1.3%. Thereby, the patient was successfully treated after methylene blue injection, associated with supportive and symptomatic management of the acquired methemoglobinemia due to indoxacarb poisoning. On day four, he was discharged from the hospital with no sequelae.

**Patient perspective:** the patient and his family were satisfied with the health care we provided during the hospitalization, and they were hopeful about his health outcomes.

**Informed consent:** the patient was informed about the publication of the case report and why the authors wanted to report it. A consent was obtained from the patient for the picture and the clinical data to be published in the journal.

## Discussion

Indoxacarb is a new oxadiazine insecticide useful in killing insects that developed resistance to pyrethroid, organophosphate, and carbamate insecticides [3]. It negatively affects the functioning of the insect nervous system by blocking voltage-gated sodium channels and generates nerve function impairment, paralysis, suspension of feeding, and death of insects [2,3]. Even though, indoxacarb is considered an organophosphate replacement with low acute and chronic mammalian toxicity [2]. There are few cases reported in the medical literature regarding indoxacarb poisoning in humans. Methemoglobinemia, rhabdomyolysis and acute kidney injury are reported as possible complications of indoxacarb poisoning [4]. In our patient, the only complication observed was methemoglobinemia. Methemoglobin is an oxidized derivative of hemoglobin. It is generated by oxidization of the oxygen-carrying ferrous iron (Fe2+) of heme to the ferric iron (Fe3+) which causes a particular bluish colour similar to cyanosis [4,5]. Each day, less than 3% of hemoglobin is naturally oxidized to methemoglobin [6]. The ferric hemes of Methb are unable to bind oxygen. The methemoglobin shifts the oxygen dissociation curve to the left leading to increased oxygen affinity for oxygen, thus impairment in the delivery of oxygen to tissues [6,7]. Decreasing enzymatic reduction of intrinsic methemoglobin is responsible for congenital methemoglobinemia, whereas exposure to drugs or toxins causes anoxidation of hemoglobin and leads to acquired methemoglobinemia [6].

The symptoms of methemoglobinemia are vague, and they depend on MetHb levels. The symptoms usually increase when MetHb levels increase [2]. For a level between 10-20%, the patient usually presents with only cyanosis [4]. Symptoms such as tachypnea, chest pain, tachycardia, and headache could be manifested beyond 20%. Above 40%, of neurological symptoms such as confusion, seizures, lethargy, and coma were noticed associated with cardiac manifestations like hypertension and arrhythmia [1,2]. MetHb level of more than 70% is extremely fatal and frequently leads to death [8]. In our patient methemoglobin level was 12.5%, and he had general cyanosis, coma, and hypoxemia without any other complications. Methemoglobinemia is usually diagnosed based on clinical features and history. The presence of cyanosis, hypoxemia refractory to supplemental oxygen therapy, and SpO_2_ significantly different from the SaO_2_ ("saturation gap") supports the diagnosis [3,7,8]. A saturation gap of more than 5% between SpO_2_ and SaO_2_ is a diagnostic key to the presence of MetHb [9]. Otherwise, the muddy brown discoloration of freshly drawn blood is a crucial characteristic. The confirmatory test consists of blood gas with co-oximetry that can measure different types of hemoglobin and determine the blood MetHb level [1].

Our patient was clinically cyanotic under FiO_2_100% with a chocolate brown colour of blood. His SpO_2_ of 80% was significantly different from the oxygen saturation (SaO_2_100%) and PaO_2_ (450 mmHg) in ABG analysis. The most effective antidote for toxic methemoglobinemia is the methylene blue which acts as an exogenous cofactor. It is converted to leucomethylene blue which in turn can reduce methemoglobin using NADPH reductase [7]. Methylene blue is commonly indicated for symptomatic patients, especially those with hypoxemia, or for asymptomatic patients with methemoglobin level above 20% [3]. The treatment consists of administration of 1-2 mg/kg body weight of methylene blue given intravenously in normal saline over 5-30 minutes [10]. In this case, the patient was drowsy then he presented coma, severe hypoxemia, and cyanosis with chocolate brown blood, so intravenous methylene blue at the dose of 1.5 mg/Kg (100 mg) was slowly injected for 30 minutes. Generally, the response is obtained within 1 hour. As illustrated in our patient, methylene blue minimizes the elimination half-life of methemoglobinemia to 90-120 minutes [1]. Based on the present recommendations, methemoglobin levels should be measured 1 hour after injection. A repeat dose may be necessary if the patient is still symptomatic and levels remain high [1]. At doses greater than 7 mg/kg after repeated doses, clinicians should be aware of the fact that methylene blue is itself an oxidant and may cause anemia, and acute hemolysis and may paradoxically worsen the methemoglobinemia [4].

Most patients require only one dose, as observed in our patient, who was improved with a single dose. As reported by Wu *et al*. [2], in acute indoxacarb poisoning, the patient required only one dose of intravenous methylene blue, and he recovered without complications. Even though, in some cases, repeated administration of methylene blue might be necessary. In a case reported by DK Lee *et al*. [3], multiple doses of methylene blue were administered because the patient persisted symptomatic for 23 hours with headache, cyanosis, and dyspnea. The administration of methylene blue can cause different adverse events in some patients and needs to be explored, especially in those with underlying enzyme deficiencies, such as glucose 6 phosphate dehydrogenase deficiency [9]. Ascorbic acid or vitamin C, a powerful antioxidant agent, is frequently used as an alternative therapy in cases where methylene blue is not possible, ineffective, or contraindicated. Hyperbaric oxygen and exchange transfusion are other modalities of management that have proved to be beneficial in severe cases [8,9]. Thus, as demonstrated in this case, indoxacarb is also a dangerous and toxic insecticide in humans. Here, we report the first case of indoxacarb poisoning in Tunisia. As illustrated in this case, methemoglobinemia secondary to indoxacarb ingestion can be successfully managed with methylene blue injection, associated to supportive care and symptomatic treatment. In front of the easy availability and the frequent use of indoxacrab, the physicians working in the intensive care unit and emergency department should be able to early recognize and adequately manage it, in order to have a good prognosis.

## Conclusion

Methemoglobinaemia can be a potentially life-threatening manifestation of indoxacarb poisoning. Sudden onset of cyanosis with low SpO_2_ on pulse oximeter that does not improve with an increased FiO_2_, normal PaO_2_ on ABG, and abnormal brownish coloration of the blood are the red flag signs of methemoglobinemia. Early detection and prompt management will prevent significant morbidity and mortality with a good prognosis. Methylene blue has proven to be the treatment of choice.
